# Visualization of Acute Liver Damage Induced by Cycloheximide in Rats Using PET with [^18^F]FEDAC, a Radiotracer for Translocator Protein (18 kDa)

**DOI:** 10.1371/journal.pone.0086625

**Published:** 2014-01-23

**Authors:** Akiko Hatori, Joji Yui, Lin Xie, Tomoteru Yamasaki, Katsushi Kumata, Masayuki Fujinaga, Hidekatsu Wakizaka, Masanao Ogawa, Nobuki Nengaki, Kazunori Kawamura, Ming-Rong Zhang

**Affiliations:** Molecular Imaging Center, National Institute of Radiological Sciences, Chiba, Japan; University of Birmingham, United Kingdom

## Abstract

Liver damage induced by drug toxicity is an important concern for both medical doctors and patients. The aim of this study was to noninvasively visualize acute liver damage using positron emission tomography (PET) with *N*-benzyl-*N*-methyl-2-[7,8-dihydro-7-(2-[^18^F]fluoroethyl)-8-oxo-2-phenyl-9*H*-purin-9-yl]acetamide ([^18^F]FEDAC), a radiotracer specific for translocator protein (18 kDa, TSPO) as a biomarker for inflammation, and to determine cellular sources enriching TSPO expression in the liver. A mild acute liver damage model was prepared by a single intraperitoneal injection of cycloheximide (CHX) into rats. Treatment with CHX induced apoptosis and necrotic changes in hepatocytes with slight neutrophil infiltration. The uptake of radioactivity in the rat livers was measured with PET after injection of [^18^F]FEDAC. The uptake of [^18^F]FEDAC increased in livers damaged from treatment with CHX compared to the controls. Presence of TSPO was examined in the liver tissue using quantitative reverse transcriptase-polymerase chain reaction and immunohistochemical assays. mRNA expression of TSPO was elevated in the damaged livers compared to the controls, and the level was correlated with the [^18^F]FEDAC uptake and severity of damage. TSPO expression in the damaged liver sections was mainly found in macrophages (Kupffer cells) and neutrophils, but not in hepatocytes. The elevation of TSPO mRNA expression was derived from the increase of the number of macrophages with TSPO and neutrophils with TSPO in damaged livers. From this study we considered that PET imaging with [^18^F]FEDAC represented the mild liver damage through the enhanced TSPO signal in inflammatory cells. We conclude that this method may be a useful tool for diagnosis in early stage of acute liver damage.

## Introduction

Medical drugs and their metabolites are believed to have caused liver dysfunction in many cases [1]. Jaundice appears in cholestatic liver disease; however, accurate symptoms in real-time are not easy to observe in cases of hepatocellular injury-type drug-induced liver damage. Moreover, taking a drug continuously without noticing liver dysfunction sometimes causes hepatic failure [2]. In order to inhibit deterioration, early detection and a timely cure are required. At present, evaluation of serum levels of liver-associated enzymes such as alanine aminotransferase (ALT) and aspartate aminotransferase (AST) is important, but a histological evaluation of liver damage may be difficult solely on the basis of serum biomarker measurements [2]–[4]. Because of the limitations of liver biopsy and the currently available noninvasive imaging techniques, such as computed tomography (CT) and magnetic resonance imaging (MRI), new noninvasive modalities that accurately and rapidly distinguish liver damage and assess damage severity have become a requirement in hepatology [5]. Compared to serum tests and histological assessments, positron emission tomography (PET) imaging with a specific radiotracer enables direct, quantitative, and multispatial visualization of physiological and cellular processes at multiple time points and at the macroscopic level [6].

Researchers have investigated the pathophysiological mechanisms of liver failure in liver damage models, including inflammation, apoptosis, and fibrosis [7]–[9]. A single administration of protein synthesis inhibitor, cycloheximide(CHX) has been shown to induce apoptosis [10]–[12] and necrotic changes in hepatocytes with slight neutrophil infiltration in rats [12]. Kupffer cells are resident liver macrophages and can phagocytose apoptotic hepatocytes [13]. After the engulfment of apoptotic cells in pathophysiologic conditions, activated Kupffer cells may cause damage to hepatocytes by releasing inflammatory cytokines or by inducing neutrophil infiltration [14]–[16]. Kupffer cells also produce anti-inflammatory cytokines such as interleukin-10 and play an important role in counteracting the effect of proinflammatory cytokines and attenuating liver damage [17].

It has been found that translocator protein (18 kDa; TSPO) increases in inflammatory cells during the occurrence and progress of inflammation [18]. TSPO is a receptor complex primarily present in the mitochondria. TSPO has broad functions related to the regulation of cholesterol transport, steroid hormone synthesis, porphyrin and heme transport, apoptosis, cell proliferation, anion transport, mitochondrial function regulation, immunomodulations, and inflammation [18]. Steady-state mRNA levels of TSPO expression are low in the liver and brain, and high in the adrenal glands, kidneys, spleen, skeletal muscle, and lungs [19]. TSPO expression in blood cells has also been reported, in which phagocytic cells, such as monocytes and polymorphonuclear neutrophils, show significantly higher TSPO expression than lymphocytes [20]. Thus, TSPO has become a useful biomarker for monitoring inflammation using PET with a specific radiotracer [21]. We have used PET with *N*-benzyl-*N*-methyl-2-[7,8-dihydro-7-(2-[^18^F]fluoroethyl)-8-oxo-2-phenyl-9*H*- purin-9-yl]acetamide ([^18^F]FEDAC), a TSPO-specific radiotracer, to noninvasively monitor various inflammatory diseases, such as neuroinflammation [22], lung inflammation [23], and non-alcoholic fatty liver disease [24].

In the present study, we used PET with [^18^F]FEDAC to visualize acute liver damage in a rat model prepared by injecting CHX into rats. We measured the liver uptake of [^18^F]FEDAC with a small-animal PET scanner at several time points after CHX treatment. Presence of TSPO in the liver tissue was examined under the protein level and the relationship between TSPO expression and [^18^F]FEDAC uptake was elucidated. The objective of this study was to determine the utility of [^18^F]FEDAC-PET in monitoring the occurrence and progression of acute liver damage.

## Materials and Methods

### Production of [^18^F]FEDAC

[^18^F]FEDAC (n = 10) was prepared in-house by reaction of a precursor with [^18^F]fluoroethyl bromide, with >98% radiochemical purity and 140–210 GBq/µmol specific activity [22].

### Study Animals

Male Sprague-Dawley rats (6–7 weeks old) were purchased from Japan SLC (Shizuoka, Japan). All animals received humane care and all experiments were approved by the Animal Ethics Committee of the National Institute of Radiological Sciences (Permit Number: 10–1005, Chiba, Japan) and were carried out according to the recommendations of the Committee for the Care and Use of Laboratory Animals, National Institute of Radiological Sciences.

### Preparation of a Rat Model of Liver Damage

After acclimation, 32 of 35 healthy rats were selected for the CHX treatment. The rats were anesthetized with 5% (v/v) isoflurane and injected intraperitoneally with 5 mg/kg of CHX (Sigma-Aldrich, St. Louis, MO) dissolved in 1.0 ml of physiological saline solution. They were divided into 4 groups of 8 rats each: CHX 1 h, 2 h, 4 h, and 6 h. For the control group, an additional 8 normal rats were used without CHX treatment. Four rats from each group were used to perform the small-animal PET study, and the others were sacrificed at each designated time point after CHX treatment for serum assessment and pathological examinations. Blood was drawn from the inferior vena cava of each anesthetized rat. A part of each rat liver was excised and fixed in 10% neutral buffered formalin for histopathological evaluation. The remaining liver samples were stored at -80°C for quantitative reverse-transcription polymerase chain reaction (qRT-PCR) assay and immunohistochemical examination.

### Analysis of Liver Enzymes in Serum

Blood samples were kept at room temperature for 1 h, followed by centrifugation at 3000 rpm for 15 min to prepare sera. The serum activities of alanine aminotransferase (ALT) and aspartate aminotransferase (AST) were determined using the Japan Society of Clinical Chemistry reference method according to the manufacturer’s instructions.

### Histopathology

The fixed liver samples were embedded in paraffin and cut into 4-µm-thick sections for staining with hematoxylin-and-eosin (H&E) according to a standard protocol. Frozen liver samples were cut in-house into 6-µm-thick slices by a cryotome (HM560, Carl Zeiss, Oberkochen, Germany) for immunohistochemical examinations.

### PET Study on Rats

PET scans were performed using a small-animal Inveon PET scanner (Siemens, Knoxville, TN), which provides 159 transaxial slices with 0.796 mm (center-to-center) spacing, a 10 cm transaxial field of view, and a 12.7 cm axial field of view. Before scanning, rats were anesthetized with 5% (v/v) isoflurane and maintained thereafter by 1–2% (v/v) isoflurane. Emission scans were acquired for 30 min in 3-dimensional (3D) list mode with an energy window of 350–750 keV, immediately after intravenously injecting [^18^F]FEDAC (10 MBq/0.16–0.25 mL, 40–80 pmol) into the rats (n = 4 for each group). After the PET scans were finished, all rats were sacrificed and radioactivity concentrations in the liver and blood were measured using a 1480 Wizard Automatic Gamma Counter (PerkinElmer, Waltham, MA).

All list-mode acquisition data were sorted into 3D sinograms, which were then Fourier re-binned into 2D sinograms (frames×min: 2×0.5, 3×1, 8×2, 2×5). Dynamic images were reconstructed with filtered back-projection using a Hanning filter, and a Nyquist cutoff of 0.5 cycles/pixel. A region of interest was placed on the liver using ASIPro VM (Analysis Tools and System Setup/Diagnostics Tool, Siemens Medical Solutions USA). Regional uptake of radioactivity was decay-corrected to the injection time and expressed as the standardized uptake value (SUV), which was normalized to the injected radioactivity and body weight. SUV = (radioactivity per milliliter tissue/injected radioactivity)×gram body weight. The areas under the time-activity curves (AUC_0–30 min_, SUV×min) for liver were calculated from 0 min to 30 min after injection.

### Immunohistochemical Staining Assay

Double staining with primary antibodies was performed using a rabbit anti-mouse TSPO antibody (NP155, 1∶1000) [25] and either a monoclonal mouse anti-rat ED1 antibody (1∶100; AbD Serotec, Raleigh, NC) for identifying Kupffer cells and macrophages in liver, or a monoclonal mouse anti-myeloperoxidase (MPO) antibody (1∶10; Abcam, Cambridge, MA) for identifying neutrophils. The sections were incubated with the primary antibody overnight at 4°C.

After the first immunoreaction, the sections were incubated with fluorophore-conjugated (Alexa Fluor 546 nm) secondary antibody and biotin-conjugated secondary antibody followed by the method of tyramide signal amplification (TSA) using the Fluorescein System (PerkinElmer) for 1 h at room temperature. The sections were mounted with a DAPI-containing medium (Vector Laboratories, Burlingame, CA).

The number of ED1 with TSPO-positive cells or MPO with TSPO-positive cells was quantified by manual counting in an area of 0.14 mm^2^ per field for 10 microscopic fields of the periportal or centrilobular regions in the hepatic lobule, respectively. Areas of the portal tract in the periportal region and areas of the central vein in the centrilobular region of each microscopic field were measured using Win ROOF software (Mitani, Tokyo, Japan) and counted out from each region. The results are represented as the number of ED1 with TSPO-positive cells or MPO with TSPO-positive cells per 1 mm^2^.

### RNA Preparation and Quantitative Reverse Transcriptase-polymerase Chain Reaction

Total RNA was extracted from frozen livers using Sepasol-RNA 1 Super (Nacalai Tesque, Kyoto, Japan) according to the manufacturer’s protocol. The quality of total RNA was checked by the ratio of 260/280 nm with NanoDrop ND-1000 (LMS, Tokyo). qRT-PCR was performed using the TaqMan method on an Applied Biosystems StepOne system (Carlsbad, CA). Target-specific primers and probes for signal transducers and activators of TSPO and 18S ribosomal RNA (18S rRNA) were purchased from Applied Biosystems. The normalized Ct value of each gene was obtained by subtracting the Ct value of 18S rRNA. The fold change of each gene versus the controls was calculated.

### Statistics

Data are expressed as the mean ± SE. Comparisons were made using a one-way analysis of variance followed by a Bonferroni post hoc test. The analysis was performed using GraphPad Prism 5 software (GraphPad Software, La Jolla, CA). Differences between groups were considered significant when the p-value was less than 0.05.

## Results

### Histopathology

**Figure 1:**
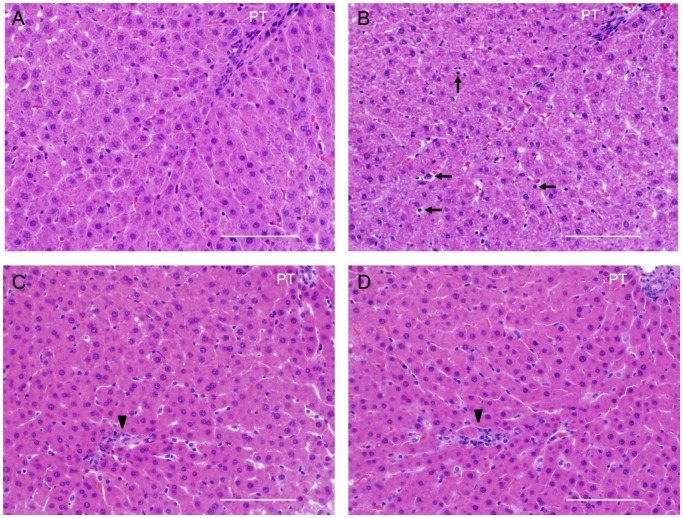
Effect of CHX treatment on histopathological images of rat livers. Hematoxylin and eosin stained sections of (A) control, (B) CHX 2 h, (C) 4 h and (D) 6 h. (B) At 2 h after CHX treatment, arrows show apoptotic cells showing marked condensation of cytoplasm and nuclear pyknosis. (C, D) Arrow heads show spotty hepatocellular necroses with mild neutrophil infiltration. Abbreviation: portal triad (PT). Scale bar: 100 µm.

Figure 1 shows H&E-stained images reflecting representative histological alterations in liver sections in the control (A), CHX 2 h (B), CHX 4 h (C), and CHX 6 h (D) groups. The control shows normal liver architecture. There was an unclear difference between the control group and 1 h of CHX treatment. After 2 h of CHX treatment, apoptotic cells showing marked condensation of the cytoplasm and nuclear pyknosis were observed in the hepatocytes. After 4 h of treatment, mild hepatocellular necroses and mild inflammatory cell infiltration were observed. Spotty hepatocellular necroses with inflammatory cells observed at 6 h were less than those seen at 4 h after treatment.

### Analysis of Liver Enzymes in Serum

**Figure 2:**
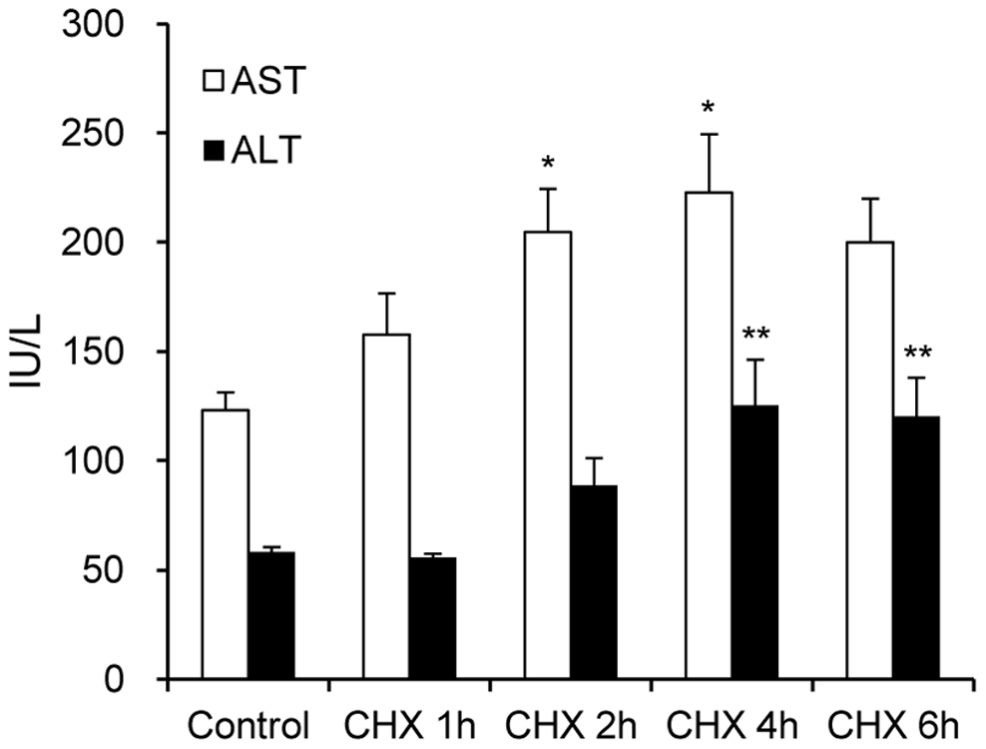
Serum ALT and AST activities. The AST and ALT activities (mean ± SE, n = 4) at 4 h after CHX treatment were 1.8 and 2.2 fold higher than those in the control group, respectively. A significant difference (p<0.05) was seen for the following comparisons, *: AST, control versus CHX 2 h or 4 h; **: ALT, control versus CHX 4 h or 6 h.

The results of serum ALT and AST activities are presented in Figure 2. The AST and ALT activities in the CHX group increased and showed the highest level at 4 h after the treatment. The AST level at 4 h, 222.8±26.7 IU/L, was 1.8 fold higher than that in the control group, 123.2±8.1 IU/L. Also, the ALT level at 4 h, 125.4±20.8 IU/L, was 2.2 fold higher than that in the control group, 58.2±2.3 IU/L. The AST and ALT activities at 6 h after CHX treatment, 200.0±19.9 and 120.3±17.7 IU/L, respectively, showed a tendency to decrease.

### PET Study

**Figure 3:**
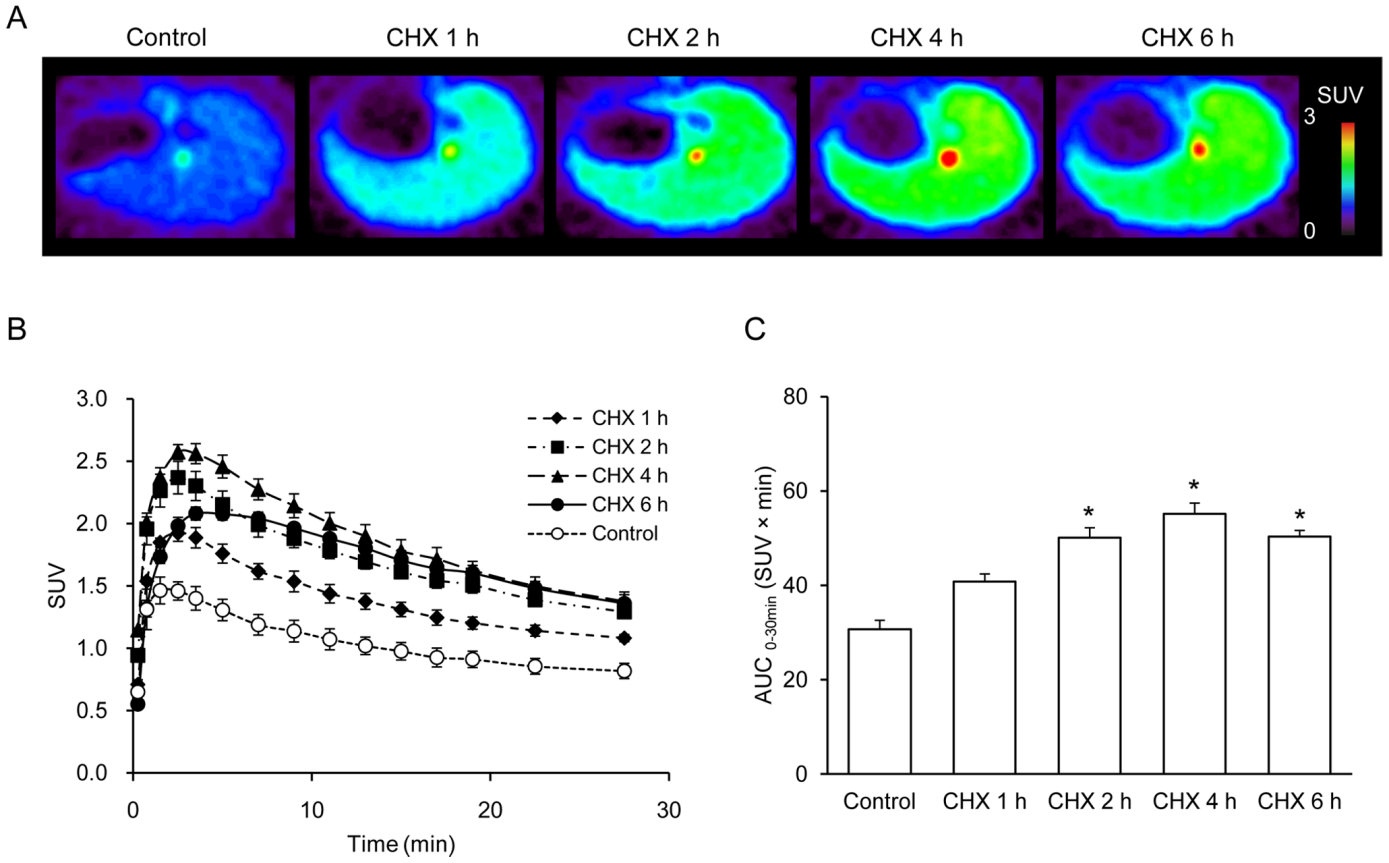
Radioactivity accumulating in the livers of the control and CHX-treated rats. (A) Representative transverse PET liver images acquired between 0 and 30 min after injection of [^18^F]FEDAC. The pseudocolor bar represents the level of [^18^F]FEDAC accumulation in the liver. The livers were more visible with time by 4 h after CHX treatment. (B) Time-activity curves of the livers. (n = 4 for each group). (C) Values of areas under the time-activity curves (AUC_0–30 min_; SUV × min, mean ± SE) were calculated from the time-activity curves between 0 and 30 min. The values were increased at 2 h (1.6 fold), 4 h (1.8 fold) and 6 h (1.6 fold) after CHX treatment compared with the control group. A significant difference (p<0.05) was seen in the following comparisons, *: control versus CHX 2 h, 4 h, or 6 h.

To visualize liver damage in the CHX-treated model, PET scans were performed for 30 min after injection of [^18^F]FEDAC. Figure 3A shows representative PET summation images between 0 and 30 min. Compared to the control image, radioactivity in the livers was increased 1 h, 2 h, 4 h, and 6 h after CHX treatment. The livers were more visible with time by 4 h after CHX treatment.

Figures 3B and C show the liver time-activity curves and uptake values, represented as AUC_0–30 min_. The uptake of radioactivity accumulating in the livers increased significantly 2 h after CHX treatment and reached a maximum at 4 h (Figure 3C). The values for 2 h, 4 h and 6 h treatment groups were 50.1±2.1 (1.6 fold), 55.2±2.2 (1.8 fold) and 50.3±1.3 (1.6 fold), respectively, compared with the control group (30.7±1.9).

Radioactivity concentrations in the liver and blood after the 30 min-PET scans were measured and are shown in Table 1. The liver uptake 1 h after CHX treatment increased significantly compared to that of the control group; however, the blood uptake was shown at a level similar to the controls. The ratio of radioactivity in the liver to that in the blood in the treated group was higher than that in the control group; 4.4±0.2 at 4 h after CHX treatment versus 3.1±0.2 in the control group, p<0.05. These results indicated that the present liver images are not largely confounded by the blood radioactivity.

**Table 1:** Radioactivity in liver and blood 30[^18^F]FEDAC.

	Radioactivity concentration (SUV)		
	Liver	Blood	Liver/Blood
Control	1.35±0.04	0.44±0.02	3.07±0.19
CHX 1 h	2.02±0.05a	0.49±0.04	4.16±0.41
CHX 2 h	2.15±0.10a	0.49±0.03	4.46±0.20b
CHX 4 h	2.13±0.08a	0.49±0.01	4.37±0.22b
CHX 6 h	2.17±0.07a	0.48±0.02	4.58±0.33b

Note. Data are presented as the mean ± SE. n = 4.

a Significantly different (p<0.05) from the control of Liver.

b Significantly different (p<0.05) from the control of Liver/Blood.

### Immunohistochemical Staining Assay

**Figure 4:**
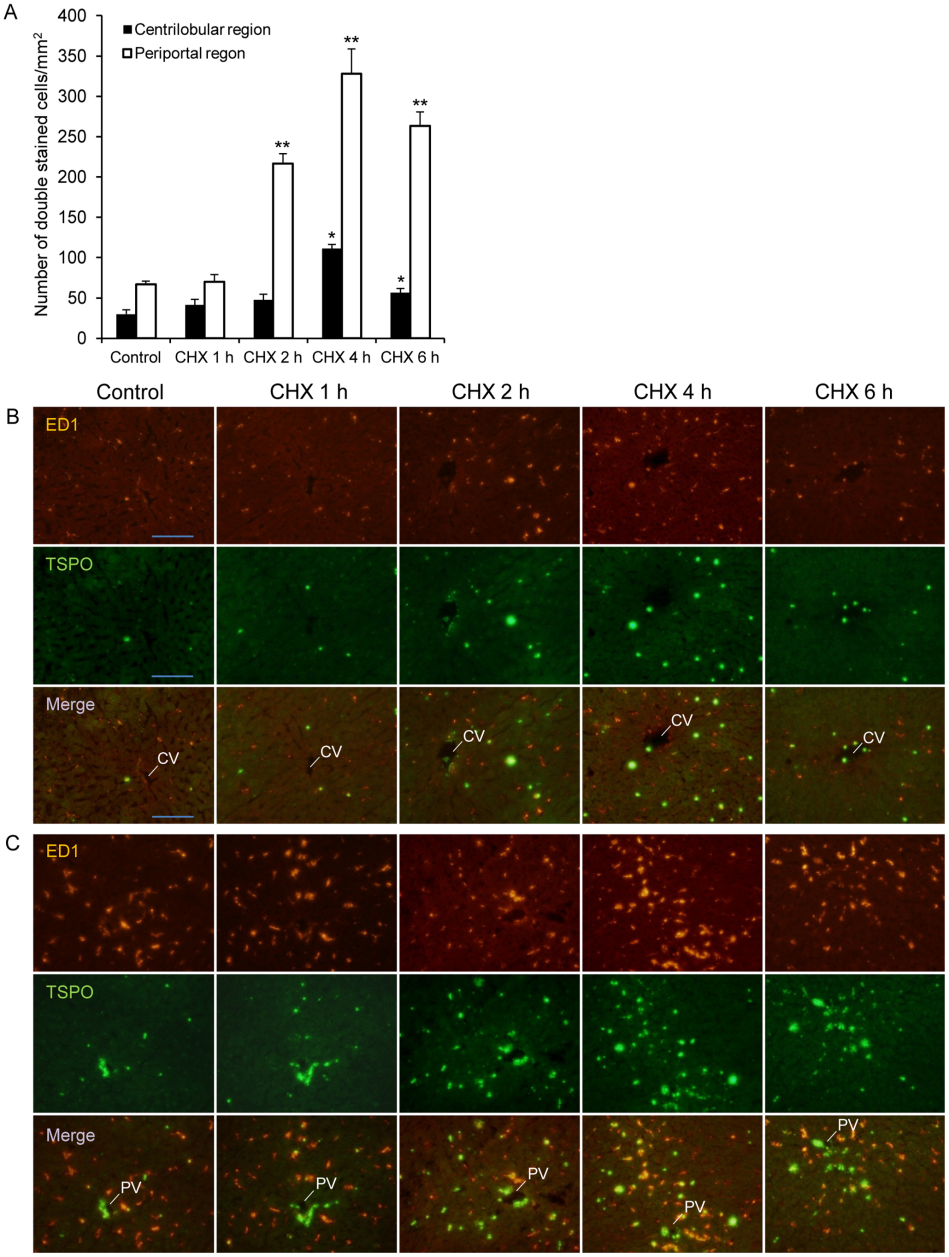
Increased TSPO levels were expressed in macrophages in liver tissues after CHX treatment. (A) The number of double immunofluorescence staining cells of ED1 and TSPO in the centrilobular and periportal regions of hepatic lobules in the control and CHX-treated rats. A significant difference (p<0.05) was seen for the following comparisons, *: centrilobular region, control versus CHX 4 h or 6 h; **: periportal region, control versus CHX 2 h, 4 h, or 6 h. (B, C) Double immunofluorescence labeling of ED1 (red) and TSPO (green) in the centrilobular region (B) and the periportal region (C). Macrophages with TSPO expression increased after CHX treatment, and higher number of double stained cells was distributed in the periportal region than in the centrilobular region at all observed time points. Abbreviations: central vein (CV), portal vein (PV). Scale bar: 100 µm.

Double immunofluorescence stainings of ED1 (red) and TSPO (green) in the centrilobular and periportal regions of hepatic lobules in the control and CHX-treated livers are shown in Figures 4B and C, respectively. In the controls, hepatic macrophages expressing TSPO, mainly comprised of Kupffer cells, were observed in a small number of cells. TSPO expression in the hepatocytes was low. The double-stained cells increased after CHX treatment both in the centrilobular and periportal regions. As shown in Figure 4C, dense fluorescence representing TSPO expression was detected in the hepatic arteriole and the intra-lobular bile duct of the portal tract, with less fluorescence in fibrotic regions, and weak signals from the portal vein were observed for all time points, including the controls. The numbers of hepatic macrophages with TSPO expression in the centrilobular and periportal regions were calculated and are shown in Figure 4A. The areas of the portal tract and central vein were counted out from the microscopic fields of the periportal and centrilobular regions, respectively. More macrophages with TSPO were distributed in the periportal region than in the centrilobular region at all observed time points. The highest number of macrophages with TSPO at 4 h after treatment was 328.0±30.7 per mm^2^ in the periportal region and 111.4±5.0 per mm^2^ in the centrilobular region.

**Figure 5:**
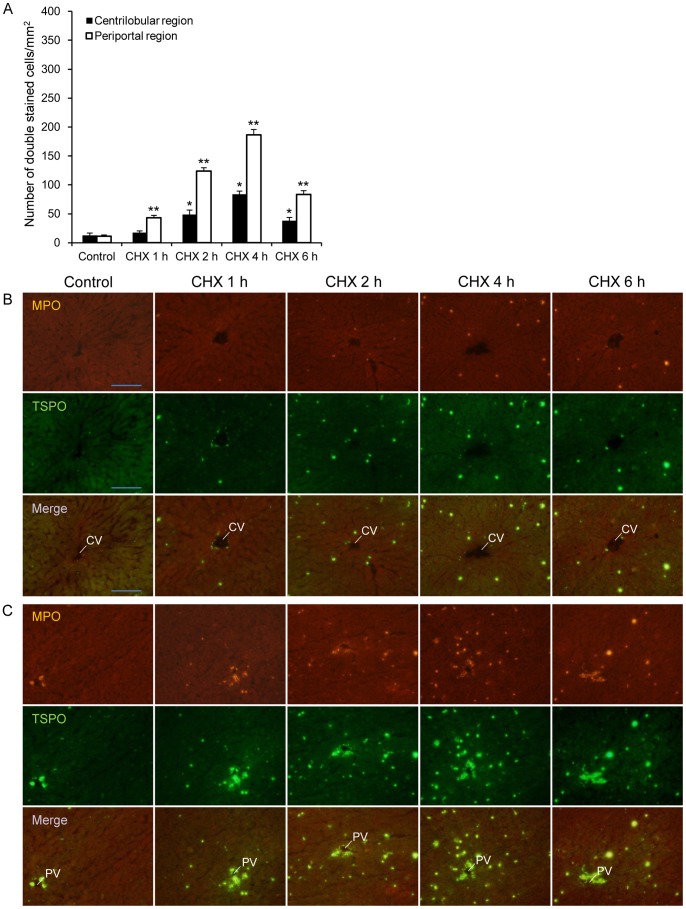
Increased TSPO levels expressed in neutrophiles in liver tissues after CHX treatment. (A) The number of double immunofluorescence staining cells of MPO and TSPO in the centrilobular and periportal regions of the rat hepatic lobules. A significant difference (p<0.05) was seen for the following comparisons, *: centrilobular region, control versus CHX 2 h, 4 h, or 6 h; **: periportal region, control versus all CHX treatment groups. (B, C) Double immunofluorescence labeling of MPO (red) and TSPO (green) in the centrilobular region (B) and the periportal region (C). Neutrophils with TSPO expression increased after CHX treatment, and higher number of double stained cells was observed in the periportal region than in the centrilobular region CHX treatment. Abbreviations: central vein (CV), portal vein (PV). Scale bar: 100< µm.

The results of MPO (red) and TSPO (green) double immunofluorescence stainings are shown in Figures 5B and C, as observed in the centrilobular and periportal regions, respectively. Neutrophils (MPO-positive cells) with and without TSPO were few in the control group. Neutrophils with TSPO expression increased after CHX treatment both in the centrilobular and periportal regions. In Figure 5C, TSPO expression in the portal tract was detected, similar to Figure 4C. The numbers of neutrophils with TSPO expression in the centrilobular and periportal regions were calculated and are shown in Figure 5A. More neutrophils with TSPO were distributed in the periportal region than in the centrilobular region after CHX treatment. The highest number of neutrophils with TSPO at 4 h after the treatment was 186.6±9.1 per mm^2^ in the periportal region and 83.8±5.4 per mm^2^ in the centrilobular region.

### Gene Expression Associated with TSPO

**Figure 6:**
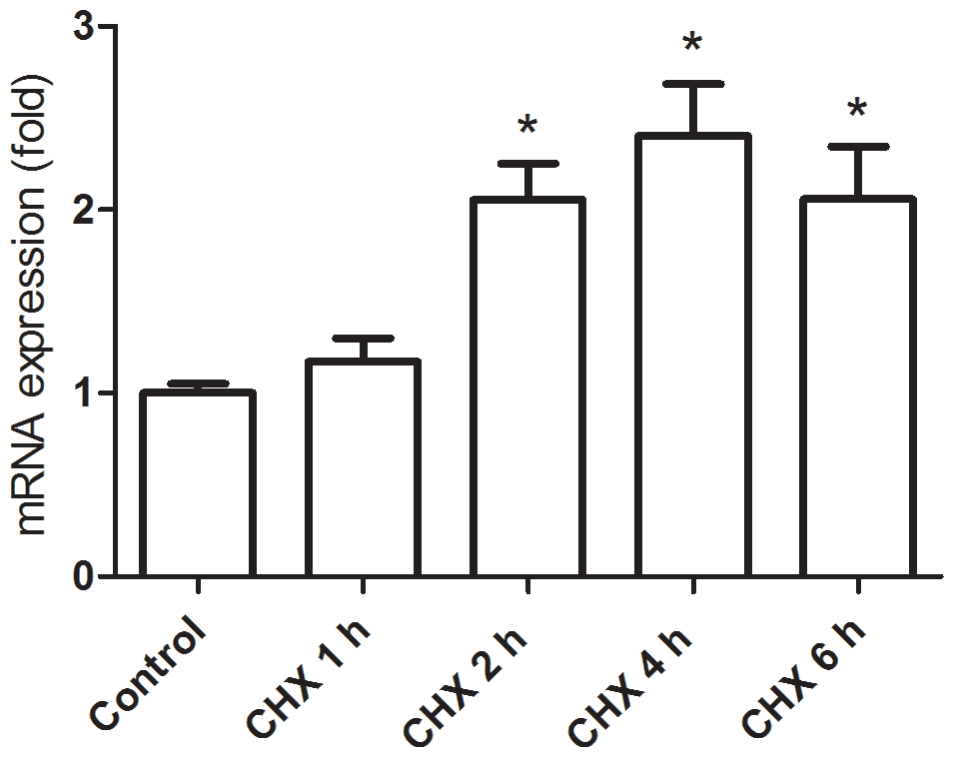
Effect of CHX treatment on the mRNA expression of TSPO. Hepatic TSPO mRNA expression was increased at 2(2.1 fold), 4 h (2.4 fold) and 6 h (2.1 fold) after CHX treatment compared with the control group. A significant difference (p<0.05) was seen in the following comparisons, *: control versus CHX 2 h, 4 h, or 6 h.

mRNA expression of the TSPO functional macromolecular signaling complex was quantified by qRT-PCR (Figure 6). Hepatic TSPO mRNA expression was increased at 2 h (2.1 fold), 4 h (2.4 fold) and 6 h (2.1 fold) after CHX treatment compared with the control group. The TSPO mRNA levels increased correspondingly with the TSPO expression and liver damage severity.

## Discussion

This study noninvasively visualized liver damage using PET with the TSPO-specific radiotracer [^18^F]FEDAC. In the PET study for the rat model of mild acute liver damage, significant increases in radioactive signals from the liver corresponded with increases in TSPO expression. The qRT-PCR and immunohistochemical assays confirmed the correlation between the increase in TSPO expression and the severity of damage. The main cellular sources of [^18^F]FEDAC uptake specific to TSPO were found to be macrophages (Kupffer cells) and neutrophils in the liver tissue.

The CHX-induced acute liver damage model was used in the present study to characterize liver changes in the early phase of liver damage. There is experimental evidence that CHX induces apoptosis and necrotic changes in hepatocytes with slight neutrophil infiltration [12]. Moreover, treatment with CHX accelerated phagocytosis of apoptotic hepatocytes by Kupffer cells and the subsequent release of inflammatory cytokines by activated Kupffer cells [13]–[16]. In addition to the histopathology experiment (Figure 1), serum chemistry assays of AST and ALT were examined in this model. Both transaminases increased to only 2-folds higher at 4 h after the CHX treatment than in the control group (Figure 2). Therefore, the present animal model was considered to have sustained mild liver damage.

The PET images with [^18^F]FEDAC for the rats at 1 h, 2 h, 4 h, and 6 h after CHX treatment reflected the occurrence and progress of liver damage (Figure 3). Comparisons of liver images and AUC_0–30 min_ values between the control and CHX-treated groups indicated that the levels of radioactivity and radiotracer binding gradually elevated in the livers along with the severity of damage. At 4 h after CHX treatment, compared to that of the control group, the AUC_0–30 min_ value of [^18^F]FEDAC increased to 1.8-fold.

The localization of TSPO in the liver was detected by immunohistochemical staining assay (Figures 4 and 5). In the controls, strong fluorescence representing TSPO expression was detected in parts of the Kupffer cells, intra-lobular bile duct, and hepatic arteriole of the portal triad, and with weak signals from the portal vein. TSPO level in hepatocytes was low regardless of CHX treatment. Our present results were consistent with the distribution patterns of TSPO immunoreactivity in human liver [26]. On the other hand, it has been reported that TSPO subcellular locations in rat liver are present not only in hepatocyte mitochondria but also in a non-mitochondrial fraction of the non-parenchymal cells with a possible biliary epithelial cell plasma membrane locations [27], although TSPO was mainly localized in the cellular mitochondria of various tissues [18]. Our present finding supports the presence of TSPO in a non-mitochondrial fraction of biliary epithelial cells, although TSPO function in the bile duct is still unknown.

The present CHX treatment induced an increase in the number of cells containing TSPO, which were expressed in macrophages and neutrophils. The activated macrophages and neutrophils in both the periportal and centrilobular regions increased and were the highest at 4 h after the treatment. The hepatic TSPO mRNA expression also indicated similar changes in activated immune cell numbers over time (Figure 6). Because the TSPO mRNA levels in phagocytic cells, such as monocytes and polymorphonuclear cells were reported to be 7–8 fold higher than that in natural killer cells and B and T lymphocytes [20], the effect of lymphocytes on the TSPO level is considered to be much less than that of macrophages and neutrophils. It was indicated that the increase in TSPO in activated macrophages and neutrophils was mainly responsible for the increase in [^18^F]FEDAC uptake in the CHX-treated livers.

Kupffer cells, the main residential macrophages in liver, are known to have a functional heterogeneity that is related to their position in the liver acinus. In the periportal region, Kupffer cells including large and heterogeneous lysosomes have high endocytic activity and high lysosomal enzyme activity. Previous studies showed that Kupffer cells are distributed over the periportal and centrilobular regions in a ratio of 1.5–2 in normal rat liver [28], [29]. Our present results showed that TSPO expressed in macrophages in control livers was 1.7-fold higher in the periportal region than that in the centrilobular region. This ratio of TSPO expression in macrophages was close to that of the whole hepatic lobule of a normal rat. On the other hand, treatment with CHX induced an increase in TSPO expressed in macrophages in the periportal region more than that in the centrilobular region, and the ratio increased to 3 at 4 h after the treatment. It is known that blood from both the portal vein and hepatic artery contained in the portal tracts of the periportal region mixes together in the sinusoids and empties into the central vein of the centrilobular region [30]. In the sinusoids, Kupffer cells are perfectly situated to clear endotoxins from the passing blood and to phagocytose debris and microorganisms. In addition, Kupffer cells also pass through the space of Disse to make direct contact with hepatocytes and to phagocytose apoptotic hepatocytes [13]. Therefore, we assume that TSPO may play a role in the function of Kupffer cells.

Acute liver injury from drug-induced liver damage, though uncommon, is of concern for both patients and medical doctors. In clinical practice, ultrasound assessment is a first line screening procedure for liver damage. However, inter-observer variability and poor reproducibility limit its usefulness. CT and MRI are more sensitive for liver damage than ultrasound. These modalities are shown in reformatted projections to fit the liver sonograms, but lack sensitivity and specificity for differentiating liver damage from other liver diseases and for assessing damage severity.

PET imaging with [^18^F]FDG, the most commonly used radiotracer, has been used to detect and characterize malignant tumors and several liver diseases [6]. However, no significant uptake of [^18^F]FDG was found in the present liver damage model and no observatory difference in liver uptake was observed between the control and CHX-treated groups (data not shown). In contrast with [^18^F]FDG, PET with [^18^F]FEDAC could noninvasively visualize liver damage by monitoring the increase in macrophages and neutrophils associated with early stages of liver damage, which could help elucidate the pathological mechanisms in liver damage induced by various drugs in the human liver. Early evaluation of liver damage is important for timely and accurate therapeutic interventions in patients suffering this disease. Therefore, using TSPO as a biomarker for PET imaging could eventually help establish a reliable and non-invasive method for monitoring liver damage. In combination with other modalities, such as serum assessment, ultrasound, CT and MRI, PET could give a reliable and accurate diagnosis of liver damage.

## Conclusion

The present findings indicate the feasibility of PET-TSPO imaging. This PET study shows excellent sensitivity and imaging quality for the detection and quantification of TSPO expression associated with the occurrence and progression of liver damage. [^18^F]FEDAC-PET appears to be a promising tool for studies involving the human liver.
